# *Chrysomya chani* Kurahashi (Diptera: Calliphoridae), a blow fly species of forensic importance: morphological characters of the third larval instar and a case report from Thailand

**DOI:** 10.1080/20961790.2018.1428078

**Published:** 2018-02-09

**Authors:** Kabkaew L. Sukontason, Tanin Bhoopat, Anchalee Wannasan, Narin Sontigun, Sangob Sanit, Jens Amendt, Chutharat Samerjai, Kom Sukontason

**Affiliations:** a Department of Parasitology, Faculty of Medicine, Chiang Mai University, Chiang Mai, Thailand; b Department of Forensic Medicine, Faculty of Medicine, Chiang Mai University, Chiang Mai, Thailand; c Institute of Legal Medicine, Forensic Biology/Entomology, Goethe-University Frankfurt, Frankfurt am Main, Germany

**Keywords:** Forensic science, forensic entomology, *Chrysomya chani*, larval morphology, identification, Thailand

## Abstract

Blow flies are worldwide the most important insects from a forensic point of view. In Thailand, aside from the two most common species, *Chrysomya megacephala* (F.) and *Chrysomya rufifacies* (Macquart), *Chrysomya chani* Kurahashi was also found to be of forensic importance. We present a case of a human female cadaver in its bloated stage of decomposition, discovered at Pachangnoi Subdistrict, northern Thailand. Entomological sampling during the autopsy displayed an assemblage of numerous dipteran larvae. Macroscopic observations showed the coexistence of third instar larvae of the three blow flies *C. megacephala*, *Chrysomya villeneuvi* Patton, an unknown blow fly species and one muscid, *Hydrotaea* sp. The minimum post-mortem interval was estimated to be six days, based on the developmental rate of *C. megacephala*. The ID of the unknown larva, which is the focus of this report, was revealed later as *C. chani* by DNA sequencing, using a 1205 bp of *cytochrome c oxidase subunit I* (*COI*). The occurrence of *C. chani* on a human body revealed the need to analyse and describe the morphology of its immature stage, to enable forensic entomologists to identify this fly species in future cases. The morphological examination of the third instar was performed, revealing peculiar characteristics: protuberant tubercles encircling abdominal segments; 9–11 lobes on the anterior spiracle; six prominent pairs of tubercles along the peripheral rim of the eighth abdominal segment; a heavily sclerotized complete peritreme of the posterior spiracles. A key to differentiate the third instar of blow flies of forensic importance in Thailand is provided.

## Introduction

Blow flies (Diptera: Calliphoridae) are insects of forensic importance, since they are the first colonizers of human cadavers, often arriving promptly after death, thereby showing a great potential in forensic investigations worldwide [1]. In Thailand, 10 blow fly species (*Chrysomya megacephala*, *Chrysomya rufifacies*, *Chrysomya villeneuvi*, *Chrysomya chani*, *Chrysomya bezziana* Villeneuve, *Chrysomya pinguis* (Walker), *Chrysomya nigripes* Aubertin, *Lucilia cuprina* (Wiedemann)*, Lucilia porphyrina* (Walker) and *Hemipyrellia ligurriens* (Wiedemann)) have been revealed to be of forensic relevance in the last decade [2–5]. *Chrysomya chani* was only reported once so far, infesting human remains in a forest [2]. Some larvae from that case were reared to the adult stage and identified by their adult morphology. Hereby, we present a second case and describe the larval morphology of this so far unknown species in forensic entomology. By providing a key for the third larval stage of *C. chani* and describing the molecular identification (of any developmental stage or even just fragment) of that species, we establish an identification tool for this species.

## Case report

### Case history

**Figure 1.**
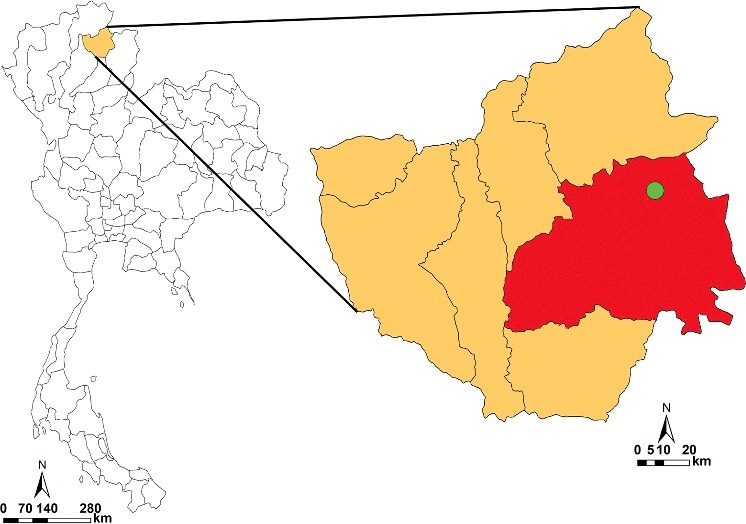
Map of Thailand showing the collection site where the human remains discovered, at Pachangnoi Subdistrict (N 19°19′24.24″; E 100°27′17.28″), Pong District, Phayao Province (green dot), northern Thailand.

In August 2006, the remains of a 38-year-old female were discovered in Pachangnoi Subdistrict (N 19°19′24.24″; E 100°27′17.28″), Pong District, Phayao Province, northern Thailand (Figure 1), and transferred to the Department of Forensic Medicine, Faculty of Medicine, Chiang Mai University. Forensic autopsy revealed the impact of blunt and sharp forces on the head and the abdomen. The bloated body was infested by fly maggots. Two blow flies, *C. megacephala* and *C. villeneuvi*, and one muscid species belonging to the genera *Hydrotaea* were identified. The minimum post-mortem interval (PMI_min_) was estimated to be six days, based on the developmental rate of *C. megacephala*. However, identification of one fly species could not be achieved due to the limited information on fly larvae morphology of species of forensic importance in Thailand at that time.

### Morphological identification

Initial macroscopic examination of the fly larvae sampled showed two groups of maggots: third instars of non-hairy maggots and third instars hairy maggots. The non-hairy maggots were identified as the blow fly, *C. megacephala* and the muscid *Hydrotaea* sp. The hairy maggots were mainly identified as third instars of the blow fly, *C. villeneuvi* [2]. Interestingly, we sampled about 20 third instars specimens that were unfamiliar to us. All larvae sampled were preserved in 70% alcohol. The largest larva was measured using Vernier calipers. In 2017, we decided to reanalyse the unidentified larvae of this case. Some specimens were cut using a sharp blade at two sites, across the third thoracic segment and across the middle of the eighth abdominal segment, to examine the cephaloskeleton and posterior spiracle, respectively. The anterior and posterior ends were transferred into a small eppendorf tube consisting 10% KOH. These tubes were then put into a beaker (half-filled with boiling water) which was placed on a hotplate (Barnstead/Thermolyne, Model: SP46920-26, USA) for 10 min. Specimens were washed twice with distilled water. To remove the alcohol, specimens were placed in Cellosolve (ethylene glycol monoethyl ether) and left for 5 min. The specimens were then transferred onto a glass slide and one drop of Euparal® was added. Specimens were arranged in their appropriate positions and covered with a cover slip. Prepared anterior and posterior ends were examined and photographed under a light microscope (Olympus CX41, Tokyo, Japan, with Olympus DP22 digital camera). For photographing, the focus stacking was shot by taking a series of images with the same composition and gradually changing the area of sharp focus. The number of pictures taken depended on the thickness of the specimen. For preserved larvae, stacking pictures were taken using a Nikon D7100 digital camera with a Nikkor lens Af-s macro 60 mm f2.8G. Each picture was merged in the program Helicon Focus 6.6.0 using method C (pyramid stacking). Terminology for general larval morphology followed Courtney et al. [6], for peripheral tubercles of larvae followed Liu and Greenberg [7] and for modifications of larval cephaloskeleton followed Szpila et al. [8].

**Figure 2.**
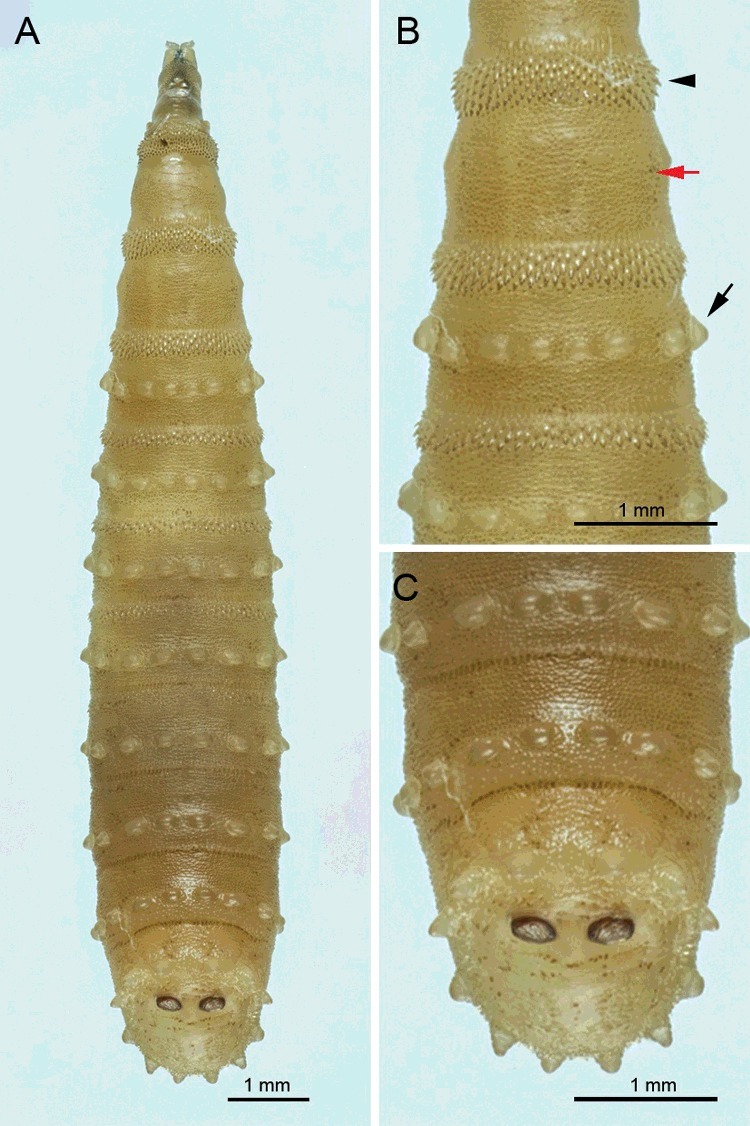
Light micrographs of third instar larvae of *Chrysomya chani*. (A) Whole body, dorsal view. (B) Anterior end showing the prominent spine bands between the second and third thoracic segments (arrowhead), and prominent tubercles encircling the body, initiating from the first abdominal segment (arrow) and denticles at the surface integument (red arrow). (C) Posterior end showing prominent tubercles encircling the body and a pair of posterior spiracle.

**Figure 3.**
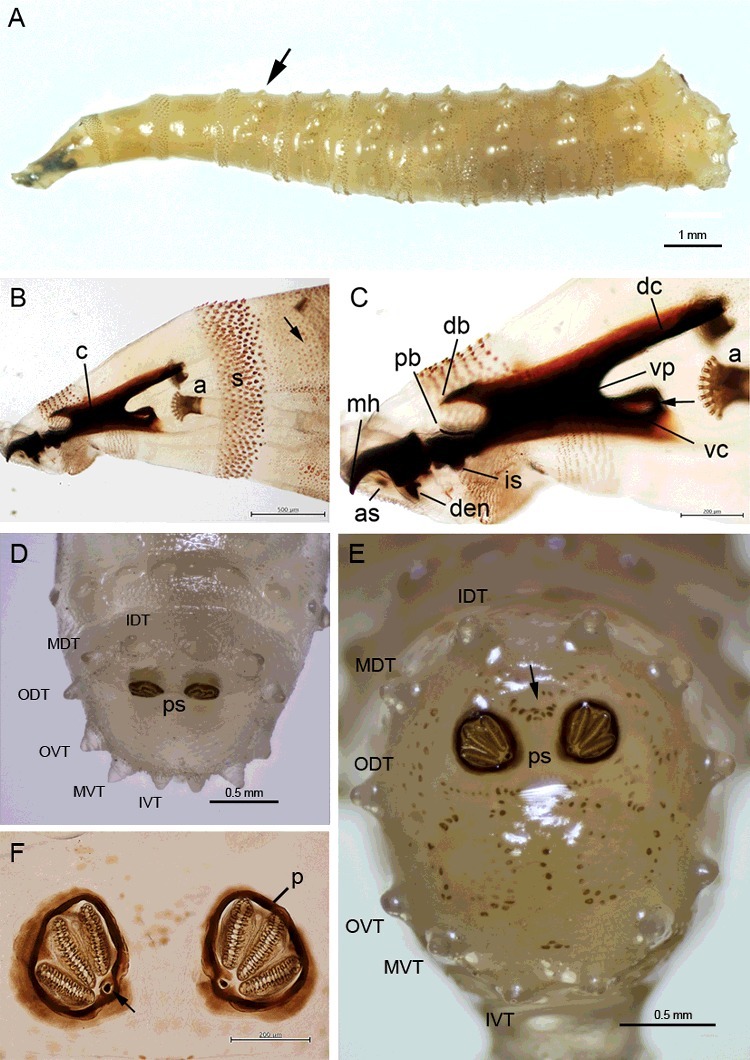
Light micrographs of third instar larvae of *Chrysomya chani*. (A) Whole body, lateral view; anterior end at left, posterior end at right. Arrows indicate the initial protuberant tubercles encircling body segments at the first abdominal segment (arrow). (B) Anterior end showing cephaloskeleton (c), anterior spiracle (a) and spine bands (s) between the first and second thoracic segments. Anterior spiracle consisted of 9–11 papillae arranging in a single row. Arrow displays surface integument, covering with dense variable size of denticles. (C) Higher magnification of the cephaloskeleton and anterior spiracle (a). Arrows indicate window of ventral cornua. Abbreviation of cephaloskeleton: as, accessory sclerite; db, dorsal bridge; dc, dorsal cornua; den, dental sclerite; is, intermediate sclerite; mh, mouthhooks; pb, parastomal bar; vc, ventral cornua; vp, vertical plate. (D) Posterior view of the eighth abdominal segment showing prominent six pairs of tubercles along the peripheral rim. Posterior spiracle (ps) is apparent. Abbreviation of tubercles: IDT, inner dorsal tubercles; MDT, median dorsal tubercles; ODT, outer dorsal tubercles; OVT, outer ventral tubercles; MVT, median ventral tubercles; IVT, inner ventral tubercles. (E) Posterior view of the eighth abdominal segment showing remarkably sculpture encircling the posterior spiracles (ps) and adjacent to these tubercles (arrow). (F) Higher magnification of posterior spiracles displaying thick, heavily sclerotize complete posterior spiracular peritreme (p) enclosing three spiracular slits. Arrow indicates indistinct button (or ecdysial scar).

The largest specimen was 12.8 mm long. The prominent features are the protuberant tubercles encircling body segments, of which the prominent tubercles originate from the first abdominal segment and are present until the eighth abdominal segment (Figures 2(A–C) and 3(A)). Spine bands between segments are obvious in the thoracic segments, with the most prominent between the first and second thoracic segments (Figure 2(A)). These spine bands between thoracic segments are relatively sclerotized (Figures 2(A,B). However, spine bands between the abdominal segments are unnoticeable (Figure 2(C)).

A very distinct ultrastructure of the surface integument is seen on the body surface, which is covered with dense variable size of denticles (Figures 2(A–C) and 3(B)). The anterior spiracle consisted of 9–11 lobes (*n* = 13) arranged in a single row (Figures 3(B,C)). The cephaloskeleton (Figure 3(C)) has large and heavily sclerotized mouthhooks, curved downwards. The posterior base of the mouthhooks is large and broad. An accessory sclerite is moderately sclerotized, adjacent to the base of the mouthhooks. The dental sclerite is apparent, curved backward apically and connected to the base of the mouthhooks. An intermediate sclerite is present. The parastomal bar is slender and slightly curved upward apically. The dorsal bridge is slender apically and bent downward, with the same length as the anterior margin of parastomal bar. The dorsal cornua, vertical plate and ventral cornua are heavily sclerotized. The dorsal cornua are much longer than the ventral cornua. The ventral cornua have an opening or window (Figure 3(C), arrow).

The posterior end of the third instar shows six prominent pairs of tubercles along the peripheral rim of the eighth abdominal segment (Figures 2(A,C) and 3(E)), of which all six pairs (inner dorsal, median dorsal, outer dorsal, outer ventral, median ventral and inner ventral tubercles) are almost equally in their protuberance. Viewed posteriorly, there is a remarkably sculpture encircling the posterior spiracles and adjacent to these tubercles (Figure 3(E)). Higher magnification of the posterior spiracles revealed thick, heavily sclerotized complete posterior spiracular peritremes enclosing three spiracular slits (Figure 3(F)). The button (or ecdysial scar) is indistinct (Figure 3(F), arrow).

**Figure 4.**
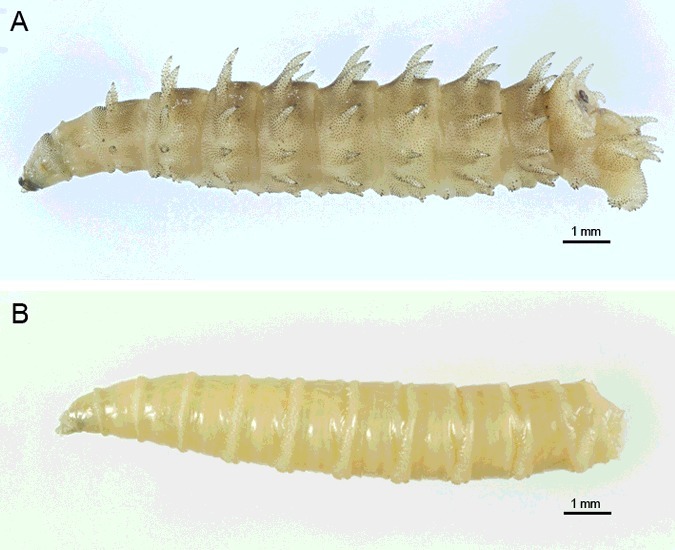
Light micrographs of third instar larva of *Chrysomya villeneuvi* and *Lucilia cuprina*. (A) Third instar larva of *C. villeneuvi* showing large, elongate tubercles on the abdominal segments. (B) Third instar larva of *L. cuprina* showing smooth abdominal segments.

**Figure 5.**
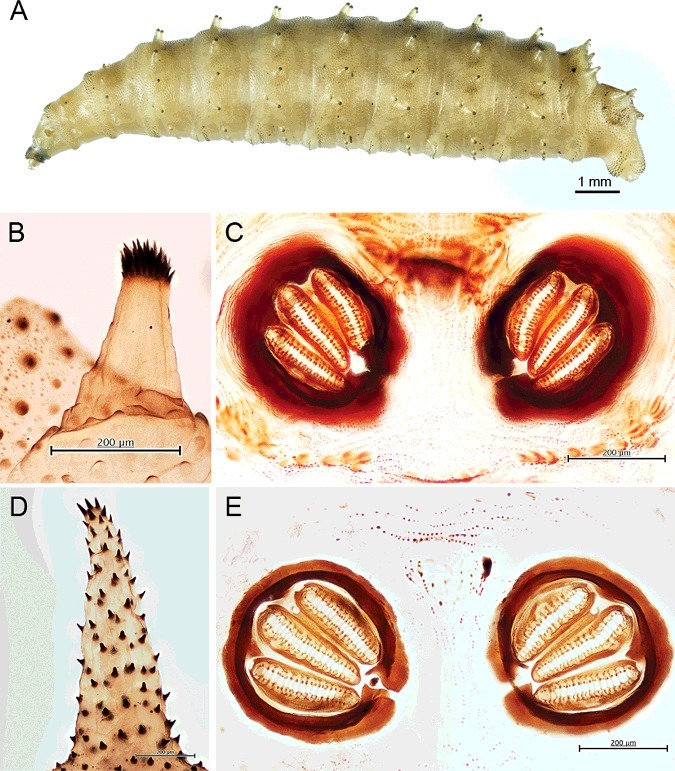
Light micrographs of larva of *Chrysomya rufifacies* and *Chrysomya villeneuvi*. (A) Third instar larva of *C. rufifacies* showing large, elongate tubercles on the abdominal segments. (B) Tubercle of *C. rufifacies* larva bears numerous small spines at tip. (C) Posterior spiracle of *C. rufifacies* showing large, heavily sclerotized incomplete peritreme. (D) Tubercle of *C. villeneuvi* larva bears numerous small spines throughout. (E) Posterior spiracle of *C. villeneuvi* larva showing large, heavily sclerotized incomplete peritreme.
  Abdominal segments lacking large, elongate tubercles (Figure 4(B))…………3
2 Tubercles bear numerous small spines at tip (Figure 5(B)); anterior spiracle with 9–12 lobes; posterior spiracle large, with heavily sclerotized incomplete peritreme (Figure 5(C))………*C.rufifacies* (Macquart)
  Tubercles bear numerous small spines throughout (Figure 5(D)); anterior spiracle with 13–15 lobes; posterior spiracle large, with heavily sclerotized incomplete peritreme (Figure 5(E))…………*C.villeneuvi* Patton
3 Abdominal segments bear protuberant tubercles (Figure 2(A–C)); anterior spiracle with 9–11 lobes; six prominent pairs of tubercles along the peripheral rim of the eighth abdominal segment (Figures 3(D,E)); posterior spiracle large, with moderately sclerotized complete peritreme (Figure 3(F))………*C.chani* Kurahashi
  Abdominal segments without protuberant tubercles…………4
4 Peritreme incomplete………5
  Peritreme complete…………7
5 End of upper peritreme gradually enlarged (Figure 6(A)); spines between the first and second thoracic segment large, multipointed (Figure 6(B)); some specimens with brown patch on dorsal integument (Figure 6(C)), but some not (Figure 6(D))……………*C.nigripes* Aubertin

A key for identification of the third instar of blow flies of forensic importance in Thailand is provided as follows:

1 Abdominal segments with large, elongate tubercles (Figures 4(A) and 5(A))…………2

**Figure 6.**
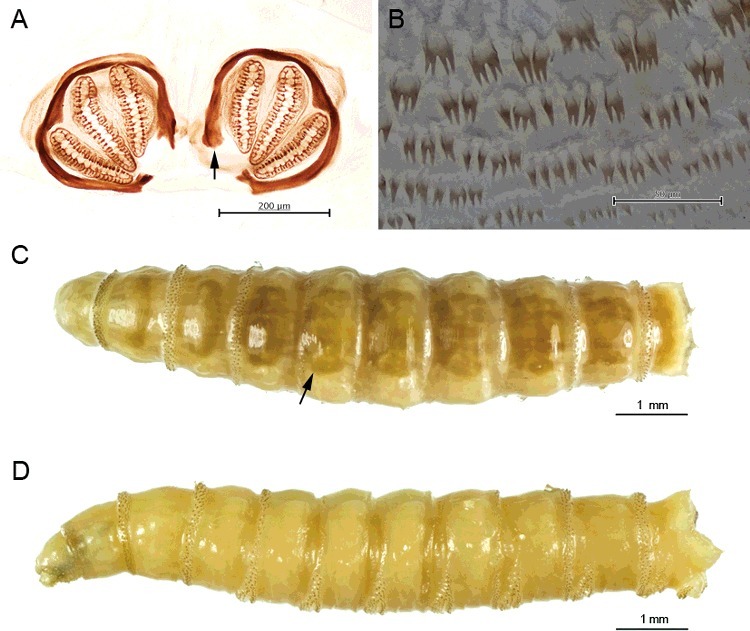
Third instar larva of *Chrysomya nigripes.* (A) Posterior spiracle showing gradually enlarged of end of upper peritreme (arrow). (B) Spines between the first and second thoracic segment. (C) A specimen with brown patch on dorsal integument (arrow). (D) A specimen without brown patch of dorsal integument.
  End of upper peritreme normal; never with brown patch on dorsal integument………6
6 Anterior spiracle with 4–6 lobes; posterior spiracle large, with moderately sclerotized incomplete peritreme (Figure 7(A)); spines between the first and second thoracic segment large, single point (Figure 7(B))…………*C.bezziana* Villeneuve

**Figure 7.**
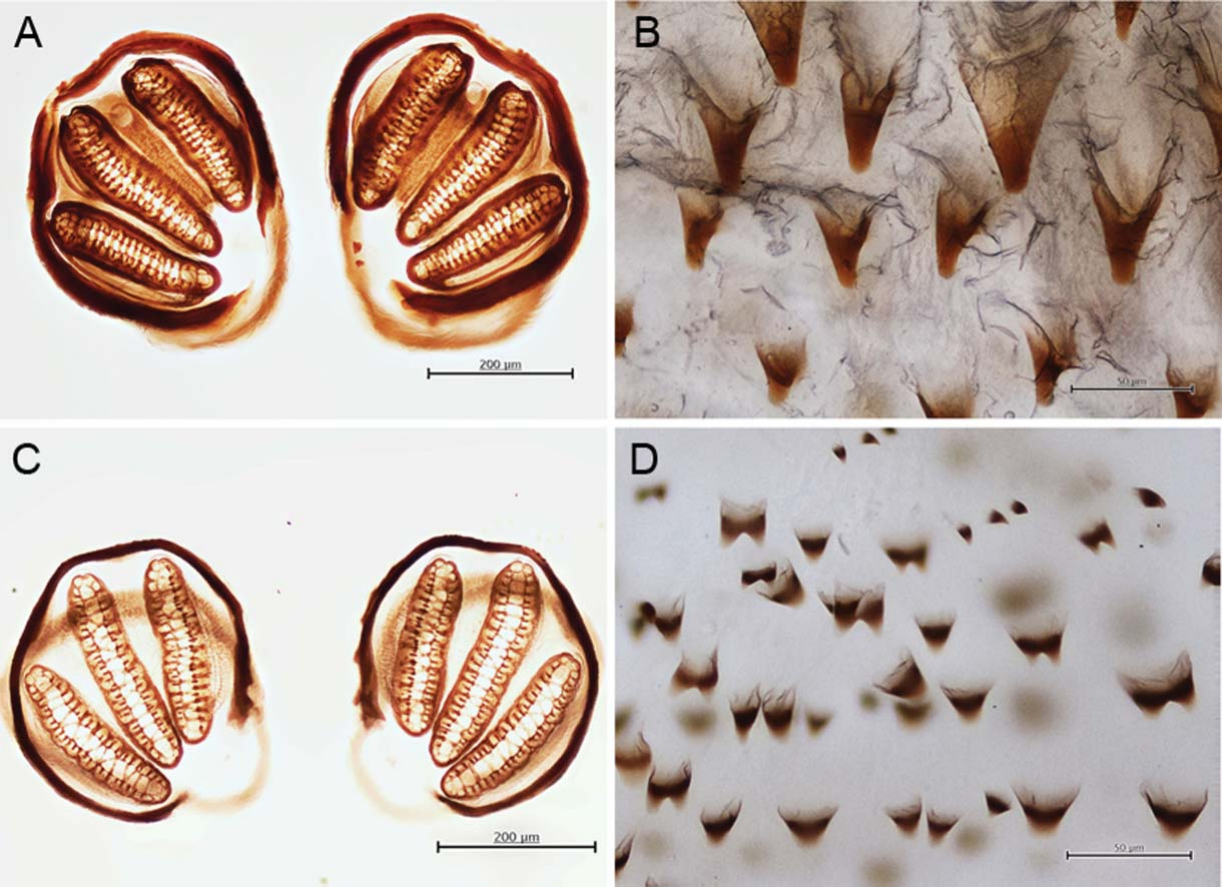
Light micrographs of larva of *Chrysomya bezziana* and *Chrysomya megacephala*. (A) Posterior spiracle of larva of *C. bezziana*. (B) Spines between the first and second thoracic segment of *C. bezziana*. (C) Posterior spiracle of larvae of *C. megacephala.* (D) Spines between the first and second thoracic segment of larvae of *C. megacephala.*
  Anterior spiracle with 9–12 lobes; posterior spiracle large, with moderately sclerotized incomplete peritreme (Figure 7(C)); spines between the first and second thoracic segment moderate, single or multipointed (Figure 7(D))…………*C.megacephala* (F.)^1.^
7 With prominent outer ventral tubercle at the rim of the eighth abdominal segment; anterior spiracle with 9–12 lobes; posterior spiracle lightly sclerotized with incomplete peritreme (Figure 8(A)); spines between the first and second thoracic segment large, arrange singly or rows (Figure 8(B)); accessory sclerite heavily sclerotized (Figure 8(C))…………*Lucilia sinensis* Aubertin

**Figure 8.**
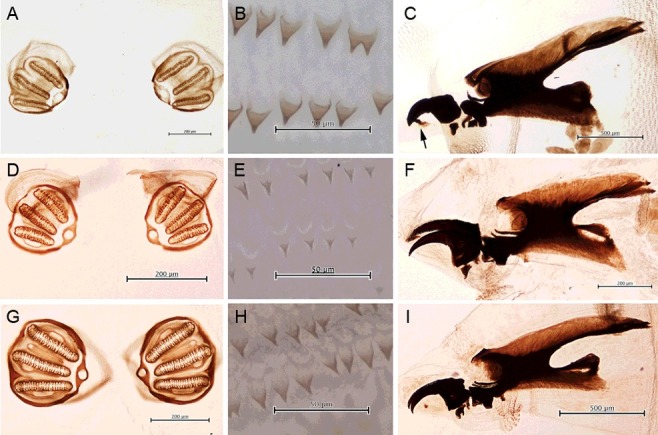
Light micrographs of larva of *Lucilia sinensis, Lucilia cuprina* and *Lucilia porphyrina*. (A–C) Larva of *L. sinensis.* (A) Posterior spiracle. (B) Spines between the first and second thoracic segment. (C) Cephaloskeleton showing accessory sclerite (arrow). (D–F) Larva of *L. cuprina*. (D) Posterior spiracle. (E) Spines between the first and second thoracic segment. (F) Cephaloskeleton. (G–I) Larva of *L. porphyrina*. (G) Posterior spiracle. (H) Spines between the first and second thoracic segment. (I) Cephaloskeleton.
  Without prominent outer ventral tubercle at the rim of the eighth abdominal segment…………8
8 Posterior spiracle small, lightly sclerotized (Figure 8(D)); anterior spiracle with 3–6 lobes; spines between the first and second thoracic segment small, arranged mostly in group or row (Figure 8(E)); accessory sclerite unsclerotized (Figure 8(F))…………*L.cuprina* (Wiedemann)
  Posterior spiracle large, moderately sclerotized (Figure 8(G)); anterior spiracle with 5–9 lobes; spines between the first and second thoracic segment small, arranged mostly in rows (Figure 8(H)); accessory sclerite unsclerotized (Figure 8(I))………*L.porphyrina* (Walker)

Specimens of larvae used in the key were from laboratory colony and/or forensic death scenes, by which adults were confirmed by morphology.

### Molecular identification

Some samples of these unknown species have been preserved in 70% ethanol since 2006 with the label of date and time of collection. To confirm the state of the species, molecular identification was performed in 2017 using these samples kept for more than 10 years.

For DNA extraction, polymerase chain reaction (PCR) amplification and DNA sequencing, the genomic DNA was extracted from one larva according to the dilution protocol of the Phire Animal Tissue Direct PCR Kit (Thermo Scientific). After measuring the DNA concentration, 100 ng/mL of the extracted DNA was subsequently used for PCR reaction.

DNA amplification was performed according to the PCR protocol of the kit. Partial *COI* sequences were amplified using the primers TY-J-1460 (5′-TACAATTTATCGCCTAAACTTCAGCC-3′) and C1-N-2800 (5′-CATTTCAAGCTGTGTAAGCATC-3′) [9]. Cycling condition was initially denaturation at 98 °C for 5 min, followed by 40 cycles of denaturation at 98 °C for 5 s, annealing at 61.7 °C for 5 s, extension at 72 °C for 30 s, followed by extension at 72 °C for 1 min. PCR products were electrophoretically separated in an 1% agarose gel, stained with RedSafe™ (Intron Biotechnology). For sequencing, the unpurified PCR products were sent to the *First BASE Laboratories Sdn Bhd* (Selangor, Malaysia).

To perform the sequence alignment and phylogenetic analysis, the obtained DNA sequences from both directions were edited and assembled using BioEdit software version 7.0.9.0. [10]. For the highest similarity search, the sequence was compared with the available sequence database via a Basic Local Alignment Search Tool (BLAST) search at the National Center for Biotechnology Information (http://blast.ncbi.nlm.nih.gov/Blast.cgi). Using MEGA6 software [11], neighbour-joining tree [12] was constructed using Kimura 2-parameter (K2P) model [13] with 1 000 bootstrap replications. Additionally, reference sequences retrieved from GenBank covering a fragment length equal or longer than ours were aligned, trimmed and added to the analyses.

**Figure 9.**
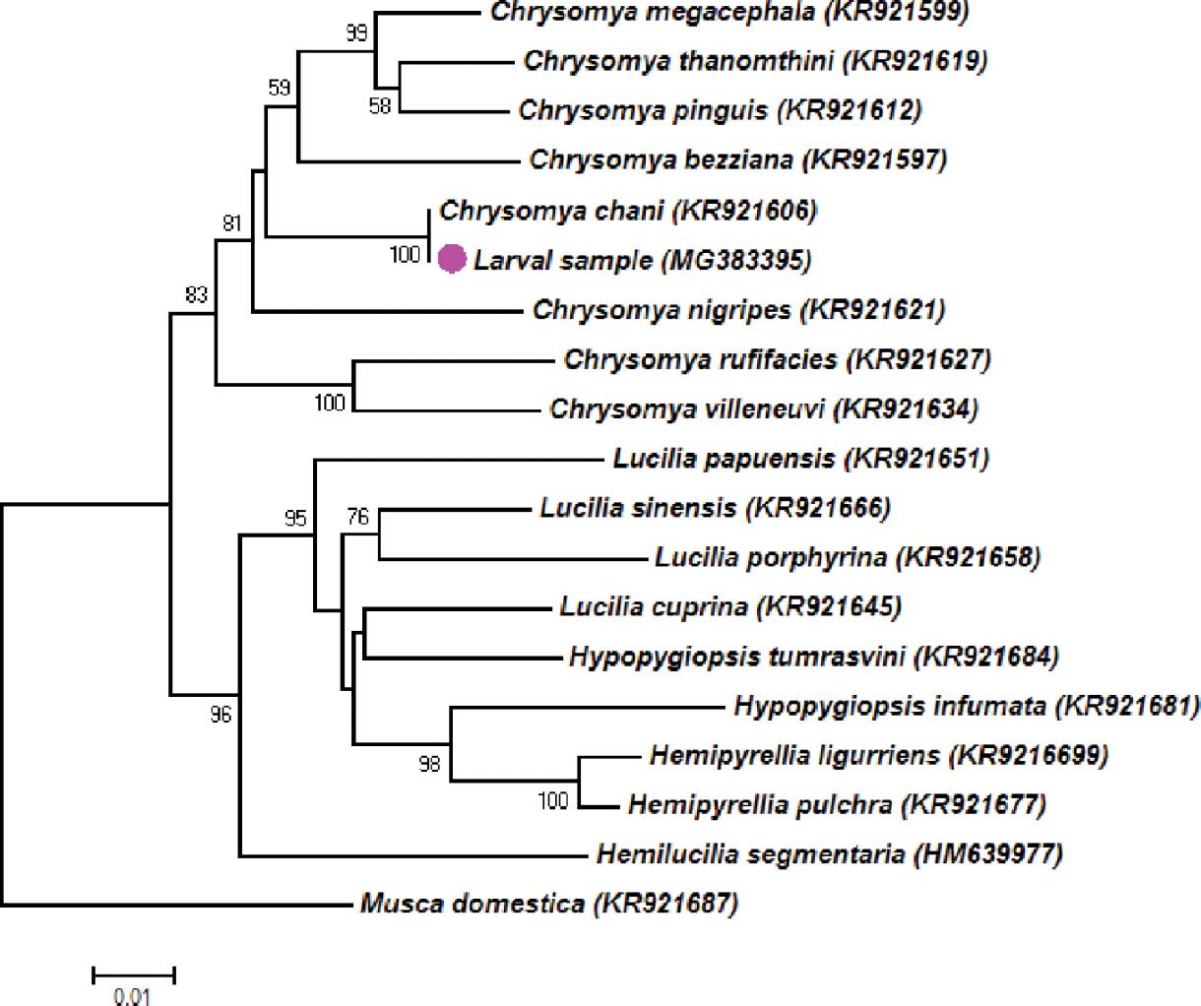
Neighbour-joining tree based on *COI* gene (1 205 bp) of *Chrysomya chani* collected from the human remain from Thailand (pink dot) and other blow fly species. Voucher codes or accession number are presented in parentheses. Bootstrap values (>50%) were shown nearby the node of the trees.

Based on the BLAST search, our larval sample collected from the human corpse was 100% identical to *C. chani* (GenBank accession no: KR921606), confirming to be *C. chani*, based on 1 205 bp of *COI* (Figure 9). Additionally, analysis of phylogenetic analysis revealed that *C. chani* was grouped within *C. megacephala*, *C. pinguis*, *C. thanomthini*, *C. bezziana* and *C. nigripes* [14].

## Discussion

To our knowledge, biological information of *C. chani* is rather limited [15,16]. This species was first described as a new species from Singapore in 1979, of which male genitalia and female ovipositor have been illustrated [15]. This is the first report on morphological characteristics of the third larval instar of *C. chani*. Altogether, morphological and molecular tools now strongly affirmed *C. chani* as a forensically important species in Thailand. It is our belief that this fly is much more common on human bodies in Asia than expected so far, and that the problem of identification is the main reason for this. The case 11 years ago illustrates this dilemma.

Several morphological characteristics of third larval instar *C. chani* were similar to those reported in third instar of the hairy maggots, *C. rufifacies* [17], *C. villeneuvi* [18] or *Chrysomya albiceps* (Wiedemann) [19], namely the protuberant tubercles which encircle the abdominal segments, the six pairs of prominent tubercles along the peripheral rim of the eighth abdominal segment, the heavily sclerotized spine bands between the thoracic segments, the sculpture of the surface integument, the number of papillae on the anterior spiracles and the heavily sclerotized peritreme of the posterior spiracles.

Remarkably, the morphology of the male genitalia of *C. chani* showed to be very similar to those of *C. megacephala*, *C. pinguis* and *C. thanomthini* Kurahashi and Tumrasvin (Sontigun et al., unpublished data). In addition, our phylogenetic analysis based on nearly full length of *COI* (Figure 9) and *cytochrome c oxidase subunit II* (*COII*) genes (Sontigun et al., unpublished data) revealed that *C. chani* was placed within the non-hairy group (*C. megacephala*, *C. pinguis* or *C. thanomthini*) even though several morphological characteristics of third instar *C. chani* were similar to third instar of the hairy maggots (*C. rufifacies*, *C. villeneuvi* or *C. albiceps*). This finding was similar to the results of Singh et al. [14], who performed the phylogenetic analysis based on 2 386 bp of combined *COI* (1 536 bp) and nuclear carbamoylphosphate synthetase genes, and Zajac et al. [20] based on the *COI* barcode region (about 700 bp). In contrast, when using 28S nuclear rRNA gene (about 1 000 bp), *C. chani* was grouped with the hairy maggot blow flies, namely *C. rufifacies*, *C. villeneuvi*, and *C. albiceps* [20]. These findings indicate that the choice of the gene to be analysed may cause variation in the outcome and the subsequent taxa arrangements. Based on the phylogenetic incongruence between the mitochondrial and nuclear genes for *C. chani*, the combination of multiple genes should be analysed to resolve the phylogenetic relationships. In the previous studies, the phylogenetic trees of the blow flies were frequently differed when constructed using different loci of genes [21–23]. Furthermore, the phylogenetic placement of taxa was found to depend on the analysed taxa, gene length and tree-building methods [14,24]. Although the single gene was sufficient to identify blow flies it is unlikely to separate some closely related species [25–29] and it cannot be used to resolve the phylogenetic relationships. Therefore, the use of multiple genes in different loci is required to enhance confidence in species identification, particularly of closely related species, and resolve the phylogenetic relationships between species of blow flies of forensic importance.

The main characters used for identification of third instar *C. chani* observed in this study (e.g. presence of accessory sclerite on cephaloskeleton, number of papillae on anterior spiracle, heavily sclerotized posterior spiracle, complete peritreme) was related to those previously described in a key for the identification of flies of forensic importance in Malaysia [16]. Interestingly, some characters observed in this study are morphologically similar to *Hemilucilia segmentaria* (F.), a blow fly species of the Central and South America. Such characters are the particular prominent tubercles along the peripheral rims of the eighth abdominal segment and the sculpture encircling the posterior spiracles (Figure 6 in [30]). Despite sharing morphological similarity in these characters, our molecular analysis of the *COI* gene (Figure 9) revealed that these two species are rather distance in genetic consideration, based on a genetic divergence of 8.6%.

*Chrysomya chani* is an endemic species in Bangladesh (Chittagong), India, China (Guangdong, Hainan I.), Indonesia (Kalimantan), Malaysia (Borneo; Sabah, Kuala Lumpur, Pahang, Perak, Selangor), Nepal, Philippines (Luzon I., Mindanao I., Palawan I., Samar I.), Singapore, Sri Lanka, Thailand and Vietnam [31,32]. In northern Thailand, the habitat of this species is mainly natural forested areas at 335–1142 m above sea level [33], while in India, adults have also been collected from secondary forests [32]. Adults are found on decomposing animal matter in tropical rain forests [34]. Research in Selangor, Malaysia using monkey (*Macaca fascicularis* Raffles) carcasses indicates that *C. chani* occurs both in outdoor and indoor scenarios. Under outdoor conditions, adults were sampled from Days 6 to 13, indicating a preference for the decomposition stages of decay and advanced decay, while at indoor sites the species was sampled from Days 4 to 30, indicating a preference for bloated to advanced decay [35]. Assessment in Malaysia, using carcasses of the New Zealand White rabbit (*Oryctolagus cuniculus* (L.)), indicated that *C. chani* adults are mainly active during the bloated stage of decomposition (Days 2–3). Second and third larval instars were sampled during active decay (Days 3–5); while the third larval instar also was collected during the advanced stages of decay (Days 6–8) [36]. PMI_min_ estimation of six days in this case correlated with previous information. Interestingly, Omar et al. [37] showed that *C. chani* females prefer to oviposit not in the natural orifices of the animal carcasses but in the fur all over the bodies. In Shenzhen, China, *C. chani* was one of the main species, along with *C. megacephala* and *C. rufifacies*, when colonizing pig carcasses in summer [38].

Despite the limitations regarding biological information of *C. chani*, our findings may have an important implication for the use of this species in forensic investigations. Morphological details of the third instar and molecular analysis in the current study will improve identification success in future and finally elucidate its real forensic relevance. Developmental rates for the immature stages are strongly needed to be helpful in forensic investigations, particularly to estimate the PMI_min_.

## Note

1. Rearing of the third instar to adult for identification is recommended to differentiate from the closely related species, *C.**pinguis.* Alternatively, a DNA analysis can be performed.
